# Centrifugally Spun Recycled PET: Processing and Characterization

**DOI:** 10.3390/polym10060680

**Published:** 2018-06-19

**Authors:** Phu Phong Vo, Hoan Ngoc Doan, Kenji Kinashi, Wataru Sakai, Naoto Tsutsumi, Dai Phu Huynh

**Affiliations:** 1 Internship Student, Kyoto Institute of Technology, Matsugasaki, Sakyo, Kyoto 606-8585, Japan; 1770195@hcmut.edu.vn; 2 National Key Lab for Polymer and Composite, Faculty of Materials Technology, HoChiMinh City University of Technology, Vietnam National University, HoChiMinh City 700000, Vietnam; hdphu@hcmut.edu.vn; 3 Doctor’s Program of Materials Chemistry, Graduate school of Science and Technology, Kyoto Institute of Technology, Matsugasaki, Sakyo, Kyoto 606-8585, Japan; ngochoandoan@gmail.com; 4 Faculty of Materials Science and Engineering, Kyoto Institute of Technology, Matsugasaki, Sakyo, Kyoto 606-8585, Japan; wsakai@kit.ac.jp (W.S.); tsutsumi@kit.ac.jp (N.T.)

**Keywords:** centrifugal spinning, forcespinning, recycled, poly(ethylene terephthalate), fibers

## Abstract

Centrifugal spinning, which is a high-productivity fiber fabrication technique, was used to produce a value-added product from recycled poly(ethylene terephthalate) (rPET). In the present study, rPET fibers, with fiber diameters ranging from submicron to micrometer in scale, were fabricated by spinning a solution of rPET in a mixture of dichloromethane and trifluoroacetic acid. The influence of the polymer solution concentration (the viscosity), the rotational speed of the spinneret, and the inner diameter of the needles on the formation and morphology and mechanical properties of the fibers were examined through scanning electron microscopy and using a tensile testing machine. The thermal behaviors of fibrous mats with various average diameters were also investigated through differential scanning calorimetry. The smoothest and smallest fibers, with an average diameter of 619 nm, were generated using an rPET solution of 10 wt % under a rotation speed of 15,000 rpm using needles having an inner diameter of 160 μm. The fibrous mats have an average tensile strength and modulus of 4.3 MPa and 34.4 MPa, respectively. The productivity and the mechanical properties indicate that centrifugal spinning is an effective technique to fabricate high-value product from rPET.

## 1. Introduction

Poly(ethylene terephthalate) (PET) is a synthetic polyester with excellent mechanical properties, including high chemical and thermal resistance [1,2]. Because of its high performance, PET has a wide range of applications, including films, textiles, molding sheets, and water containers. Although PET is a biodegradable polymer [3], bacteria that can degrade and assimilate PET are still being studied in the laboratory. Therefore, discarded PET bottles can lead to serious environmental problems, and PET waste-bottle management and recycling have become an important issue because of an increase in PET bottle consumption [4,5]. In Japan, waste PET bottles are primarily recycled by mechanical recycling, which turns postconsumer PET bottles into recycled PET (rPET) flakes to produce recycled products. For example, in 2014, in Japan, 225.2 kilotons of rPET flakes from postconsumer PET bottles were recycled to make recycled products, such as sheets, textiles, stationary, and detergent bottles [6]. Therefore, the profit involved in rPET production is relatively low. Moreover, the cost of recycling is not always economically viable because virgin PET has a low and stable cost. Therefore, there is a demand for a method by which to increase the value of rPET products. An approach to make value-added products from rPET is to turn waste PET into PET nanofibers, which can be used in filter media as a replacement for virgin PET in order to reduce the cost [7,8].

Due to their high surface-area-to-volume and high porosity, nanofibers have been applied in high-value applications, including batteries [9], sensors [10], tissue engineering [11], drug delivery [12], and filter media [13]. Among nanofiber fabrication methods, electrospinning is the most popular technique for the production of nanofibers. Recently, a number of studies have examined the production of rPET nanofibers using electrospinning. Strain et al. fabricated a nanofibrous membrane from a recycled PET/DCM/TFA solution for smoke filtration [7]. An antimicrobial recycled PET nanofibrous membrane used for water filtration was also produced by Zander et al. [8]. While electrospinning is a simple and convenient technique, the electrospinning process still suffers from several drawbacks, including low productivity and the requirement of applying a high electric field [14,15]. Centrifugal spinning (or sometimes forcespinning) (CS) is a promising nanofiber fabrication technique that overcomes many of the disadvantages associated with electrospinning [16,17]. In this process, centrifugal forces generated at high rotational speeds create and elongate the polymer jet without the requirement of high voltage, enabling the formation of conductive and nonconductive fibers of diameters ranging from micrometers to nanometers. Recently, centrifugally spun fibers have been used in applications such as tissue regeneration [18], separators [19], smart textiles [20], and drug delivery [21]. Recycled PET fibers have also been produced using centrifugal melt spinning. Zander et al. spun recycled PET fibers with a diameter of 4.56 ± 2.97 μm via centrifugal melt spinning at 300 °C [22]. However, the relatively high viscosity of melt-spun material makes it difficult to generate thin fibers. Furthermore, the repeated heating cycle in the melt spinning process causes polymer degradation, leading to inferior mechanical properties of the resulting fibers. A possible alternative favorable technique would be to carry out CS of rPET from solution, which allows for the production of smaller fibers and for controlling fiber morphology. The used solvent can be collected using a condensation system and can be reused in order to reduce the emission of solvents, which makes the product more cost effective.

In the CS process, several processing parameters, e.g., the concentration of the polymer solution, the rotational speed of the spinneret, and the size of the needles used, will affect the properties of the final products. The processing parameters should be evaluated in order to obtain fibers with a desirable morphology and desirable properties. Therefore, the purpose of the present study was to fabricate recycled PET fibers mats using CS and to evaluate the influence of these parameters on fiber formation. The correlation between different experimental parameters on the fiber properties, including mechanical and thermal properties, was also investigated in order to establish the processing condition for feasible and effective fiber production.

## 2. Materials and Methods

### 2.1. Materials

Recycled PET pellets from postconsumer PET water bottles (CR–8816) were kindly provided by Dr. Kazushi Yamada (Advanced Fibro-Science, Kyoto Institute of Technology, Kyoto, Japan). Trifluoroacetic acid (TFA) and dichloromethane (DCM) was purchased from Wako Co., Osaka, Japan. All of the chemicals were used as received without further purification.

### 2.2. Solution Preparation and Centrifugal Spinning Process

A series of polymer solutions were prepared by dissolving rPET pellets in a mixture of solvents of TFA and DCM (7:3 *w*/*w*). The mixture was mixed for 30 min using a planetary centrifugal mixer (ARE-310, Thinky Co., Tokyo, Japan) at 2000 rpm and was kept in a constant-temperature shaker (Eyela MMS-1) at 25 °C to make a homogenous solution. The viscosity of each solution was measured at least three times with a vibro viscometer (SV-1A, A&D Co., Tokyo, Japan) at room temperature (25 °C).

**Figure 1:**
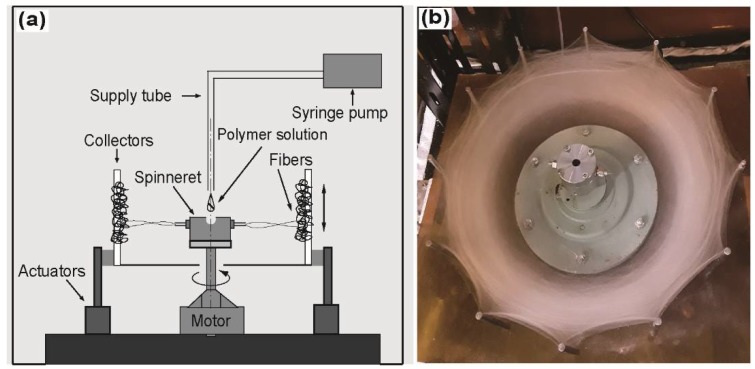
(**a**) Schematic diagram of the CS system used in the present study and (**b**) photograph of an rPET-fiber web produced by CS.

The rPET fibers were prepared using a build-up CS system of our own design based on a centrifuge machine (Tomy MC 150, Tomy Geiko Co., Tokyo, Japan). The CS system consisted of a 32-mm needle-based spinneret equipped with blunt needles with an inner diameter ranging from 60 μm to 340 μm with a shaft length of 5 mm, which was rotated by an AC motor at speeds ranging from 0 to 15,000 rpm. The feed rate of the polymer solution was controlled to 106 mL·h^−1^ by a syringe pump (KDS-100, KD Scientific Inc., Massachusetts, USA). The distance between the tip of the needles and the collectors was fixed at 10 cm. The movable collector system consisted of a linear guide rail assembly with a step motor, which was controlled by an Arduino system to collect more fibers at a wider range of orientations. The relative humidity and temperature were measured by a hygrothermograph placed inside the CS chamber and were maintained at constant values of 30% and 25 °C, respectively. A schematic diagram of the CS system and photographs of the rPET fibrous web after spinning are shown in Figure 1.

### 2.3. Characterization

The morphology of the resulting fibers was characterized using a scanning electron microscope (SEM) (S-3000N, Hitachi Co., Tokyo, Japan). The fibers were collected on the SEM sample base using conductive adhesive and were then sputter coated with a layer of gold-palladium before being measured using the SEM at an accelerating voltage of 20 kV. Image processing software (ImageJ, National Institutes of Health, Montgomery County, MD, USA) was used to measure the diameters of fibers and beads. The size distributions of the measured fibers and beads were calculated using Origin software. The diameters of the fibers were obtained by measuring 200 fibers from SEM images at a magnification of 2000×. In order to measure the bead area percentage, the samples for SEM measurements were prepared with the same basic weight of 0.5 mg·cm^−2^. The bead areas were then observed from three SEM images at a magnification of 500×. The approximate bead area percentage (BAP) was calculated using the following equation:

(1) $$BAP=\frac{S_{1}}{S_{2}}\cdot 100\%$$

where *S*_1_ and *S*_2_ are the area of the SEM image and the total area of beads in the measured SEM image, respectively.

The thermal properties of the resulting fibers were studied using differential scanning calorimetry (DSC). Thermograms were obtained using a TA Instruments Q200 calorimeter (TA Instruments Japan Inc., Tokyo, Japan) with a heat/cool/heat program. The mass of each sample of the fibrous mat, which was placed in a sealed aluminum pan, was approximately 2 mg. The sample was heated at 10 °C·min^−1^ from 30 to 300 °C under a nitrogen atmosphere at a gas flow rate of 50 μL·h^−1^, was cooled at 10 °C·min^−1^ to 0 °C, and was then heated again at 10 °C·min^−1^ to 300 °C. The degree of crystallinity ($\chi_{c}$) was calculated using the following equation:

(2) $$\chi_{c}=\frac{\left(\Delta H_{f}-\Delta H_{c}\right)}{\Delta H_{f}^{0}}$$

where $\Delta H_{f}$ is the enthalpy of fusion, $\Delta H_{c}$ is the enthalpy of cold crystallization, obtained from DSC thermogram, and $\Delta H_{f}^{0}$ = 135.8 J·g^−1^ is the enthalpy of fusion of completely crystalline PET.

The mechanical properties of rPET fibrous mats were determined using a universal tensile testing machine (TENSILON RTF–1210, A&D Co., Tokyo, Japan) (gauge length: 20 mm, crosshead speed: 0.5 mm·s^−1^, 100-N load cell). A cardboard window frame size of 30 × 40 mm, with a window size of 10 × 20 mm, was used as a mount for tensile testing. Sections of the fibrous mat were cut out and mounted on the frame using double-sided tape and were further fixed using cyanoacrylate adhesive. The samples were then stored in a desiccator for at least 24 h to remove all of the residual solvent in the fibers. The loading direction of the samples in the testing machine was parallel to the circumferential fiber collection direction. The effective cross-sectional area of the testing sample was calculated by measuring the weight of the fiber mat. Tensile tests were performed in an atmosphere of 20 °C and 30% RH. At least five samples of each fibrous mat were tested.

## 3. Results and Discussion

### 3.1. Solution Intrinsic Viscosity

In the CS process, the morphology and structure of fibers are greatly affected by the concentration/viscosity of the polymer solution used. Previous studies indicate that during the CS process, the critical entanglement concentration (*Ce*) must be exceeded in order to form detect-free fibers [23,24]. Therefore, this section focuses on the determination of the *Ce* of the polymer solution. The critical chain overlap concentration, denoted C*, was used to identify the critical entanglement concentration (*Ce*). Using the C*, the boundary separating the rheological definition of different entanglement regimes in a polymer solution can be simply demarcated [20,25]. The point at which the concentration inside a single macromolecular chain is equal to the solution concentration, or C*, can be estimated as C*~1/[η], where [η] is the intrinsic viscosity of the polymer solution.

**Figure 2:**
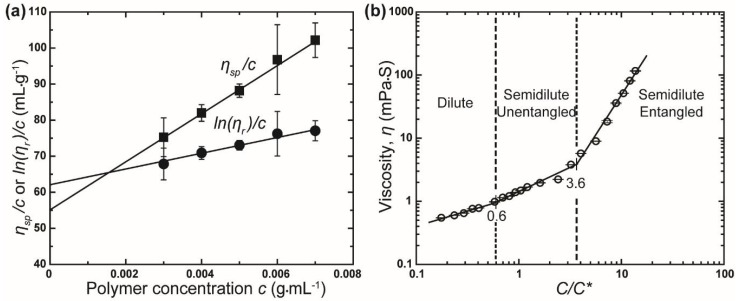
(**a**) Intrinsic viscosity of rPET solution obtained from Huggins and Kraemer equations and (**b**) scaling relationship between viscosity, *η*, and *C*/C* for various rPET solutions at 25 °C.

The intrinsic viscosity of the rPET solution was determined using the Huggins and Kraemer equations [26,27]:

(3) $$\frac{\eta_{sp}}{c}=\left[\eta \right]+\kappa_{H}{\left[\eta \right]}^{2}$$

(4) $$\;\frac{\ln\left(\eta_{r}\right)}{c}=\left[\eta \right]-\kappa_{K}{\left[\eta \right]}^{2}c$$

where $\kappa_{H}$ is the Huggins coefficient, $\kappa_{K}$ is the Kraemer coefficient, $\eta_{sp}=\left(\eta -\eta_{s}\right)/\eta_{s}$ is specific viscosity, and $\eta_{r}=\eta /\eta_{s}$ is relative viscosity (η: viscosity of the polymer solution, $\eta_{s}$: viscosity of the solvent mixture). Plots of $\eta_{sp}/c$ and $\ln\left(\eta_{r}\right)/c$ versus *c* yield two straight lines, where the intercept at *c* = 0 corresponds to the intrinsic viscosity. The twin Huggins–Kraemer plots for rPET intrinsic viscosity calculation are shown in Figure 2a. The intrinsic viscosity of the rPET solution from Huggins and Kraemer equations is 61.16 mL·g^−1^ and 54.60 mL·g^−1^, respectively. Based on these results, the average intrinsic viscosity was calculated to be 57.88 mL·g^−1^. The critical chain overlap concentration, C*, was then calculated as approximately 1/[η]. Converting this result gives a concentration of 1.2 wt % (taking the density of the mixture of solvent as 1.38 g·mL^−1^).

In the present study, zero shear viscosity was taken as the viscosity of the polymer solutions, *η*, measured using a vibro viscometer at a low frequency of 30 Hz. Based on the experimentally determined C*, the variation of the viscosity (zero shear viscosity) of different solutions as a function of concentration rescaled by C* (*C*/C*) is established, as shown in Figure 2b. The resulting plots of the viscosity values with respect to *C*/C* can be separated into different solution regimes: dilute regimes (*C*/C* < 0.6, *C* < 0.7 wt %), semidilute unentangled regimes (0.6 < *C*/C* ≤ 3.6, 0.7 < *C* ≤ 4.3 wt %), and semidilute entangled regimes (*C*/C* > 3.6, *C* > 4.3 wt %). Based on these results, the crossover of concentration from the semidilute unentangled to semidilute entangled regime occurs at *C*/C* = 3.6 (*C* = 4.3 wt %) is referred to as the critical entanglement concentration, *Ce*. Hence, rPET solutions with concentration of 5 wt % or above will be used to generate fibers and to evaluate the effect of concentration on the properties of rPET fibers.

### 3.2. Effect of Experimental Parameters on Morphology and Diameter of Fibers

**Figure 3:**
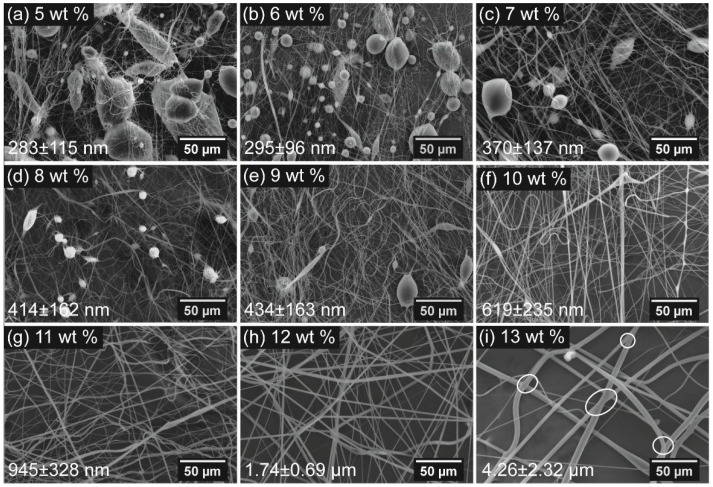
Scanning electron microscope images of centrifugally spun rPET fibers prepared from various polymer solution concentrations: (**a**) 5; (**b**) 6; (**c**) 7; (**d**) 8; (**e**) 9; (**f**) 10; (**g**) 11; (**h**) 12; and (**i**) 13 wt %. Centrifugal spinning condition: rotational speed, 15,000 rpm; needle inner diameter, 160 μm; collection distance, 10 cm.

Figure 3 shows SEM images of centrifugally spun products from various rPET solutions with the concentrations ranging from 5 wt % to 13 wt % at a rotational speed of 15,000 rpm using needles with inner diameters of 160 μm. Even though the concentration of the polymer solution exceeded the entanglement concentration, beads formed on the fiber web when the rPET content ranged from 5 wt % to 9 wt %. Smooth, bead-free fibers were observed at rPET concentrations up to 10 wt %. At concentrations above 13 wt %, the centrifugal force generated by the spinneret at a rotational speed of 15,000 rpm was insufficient to overcome the capillary force, leading to the stacking of the polymer solution inside the spinneret, so that jet formation and elongation were not obtained and fibers were not formed. The results indicated that the fibers’ morphology was not only influenced by polymer chain entanglement but was also affected by the viscoelasticity of polymer solution.

**Figure 4:**
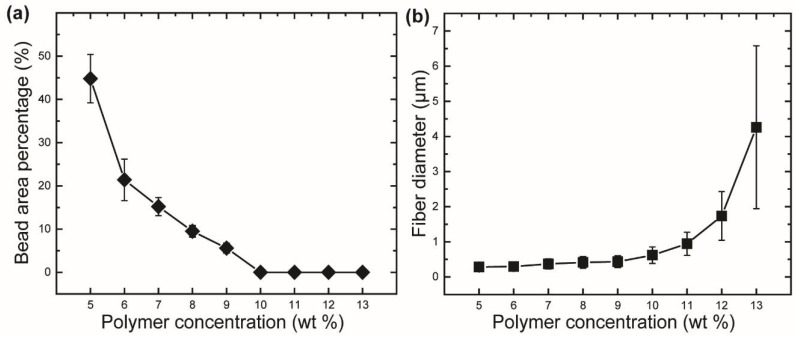
Variation of (**a**) bead area percentage and (**b**) fiber diameter of rPET fibers fabricated from various polymer concentrations. Centrifugal spinning condition: rotational speed, 15,000 rpm; needle inner diameter, 160 μm; collection distance, 10 cm.

According to Golecki et al. and Mellado et al., the appearance of beads on fibrous webs produced from low-viscosity polymer solutions is attributed to the Rayleigh instability [28,29]. When a polymer jet is spun out of needles, the geometry of the jet tends to become spherical in order to reduce the surface tension, leading to bead formation. The timescale required to form a spherical droplet is called the timescale of beading (or the timescale of the Rayleigh instability). At a low concentration (rPET concentration range from 5 wt % to 9 wt %), the relatively low viscosity leads to a decrease in the timescale of beading. During the CS process, the Rayleigh instability occurred when the solidification on the surface of the polymer jet was insufficient, resulting in the formation of beads. When the concentration reached 10 wt %, at higher viscosities, the timescale of beading of the solution was longer than the timescale of the solidification on the jet surface, which prevents the occurrence of the Rayleigh instability. Therefore, bead-free fibers were obtained at rPET concentrations of 10 wt % and above. The effect of the Rayleigh instability can be further investigated by measuring the bead area percentage (BAP), as shown in Figure 4a. The BAP decreases with the increment of the amount of polymer solution, from 44.8% ± 5.6% to 5.6% ± 1.1%, when the rPET concentration is increased from 5 wt % to 9 wt %.

When the concentration reaches 13 wt %, interconnections between the fibers are observed on the fiber mat, as indicated by the white circles in Figure 3i. With the increment of fiber diameter, the heavy weight prevents the movement of fibers, and the fibers are still wet when they are deposited on the collectors, leading to the formation of a network structure in the fiber web.

The variation of the fiber diameter with the polymer concentration was determined, as shown in Figure 4b. As shown in Figure 4b, the fiber diameter produced from a polymer gradually increases when the rPET concentrations increases from 5 wt % to 10 wt %. The average diameters are found to be 283 ± 115 nm and 619 ± 235 nm, respectively, at rPET concentrations of 5 wt % and 10 wt %. However, the fiber diameter increases significantly when the rPET concentration exceeds 10 wt % and reaches 4.26 ± 2.32 μm at a concentration of 13 wt %. The increment in the fiber diameter is attributed to the increase in the solution viscosity, which prevents the elongation of the polymer jet upon rotational spinning.

**Figure 5:**
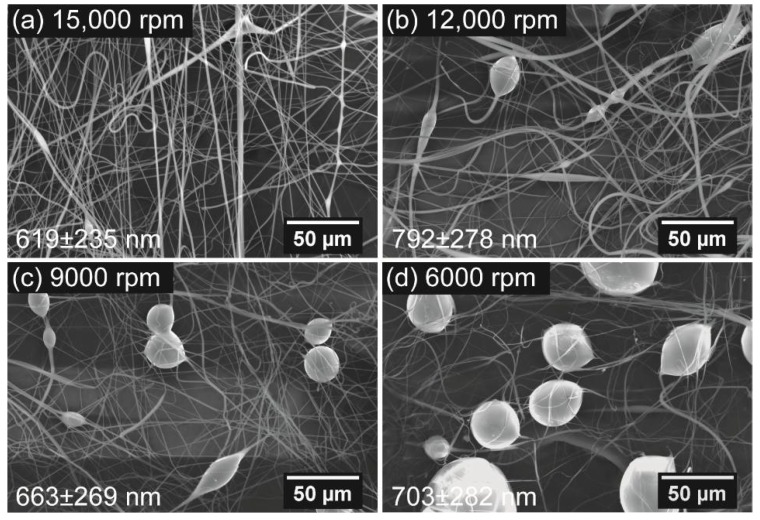
Scanning electron microscope images of centrifugally spun rPET fibers prepared under various rotation speeds: (**a**) 15,000; (**b**) 12,000; (**c**) 9000; and (**d**) 6000 rpm. Centrifugal spinning condition: polymer solution concentration, 10 wt %; needle inner diameter, 160 μm; collection distance, 10 cm.

The morphology of centrifugally spun fibers can also be affected by rotational speed. Four rotational speeds of 6000, 9000, 12,000, and 15,000 rpm were used to study the relationship between the rotational rate and the fiber morphology, using needles with an inner diameter of 160 μm and a 10-wt % rPET solution. Scanning electron microscope images of the samples fabricated from the four speeds are shown in Figure 5.

**Figure 6:**
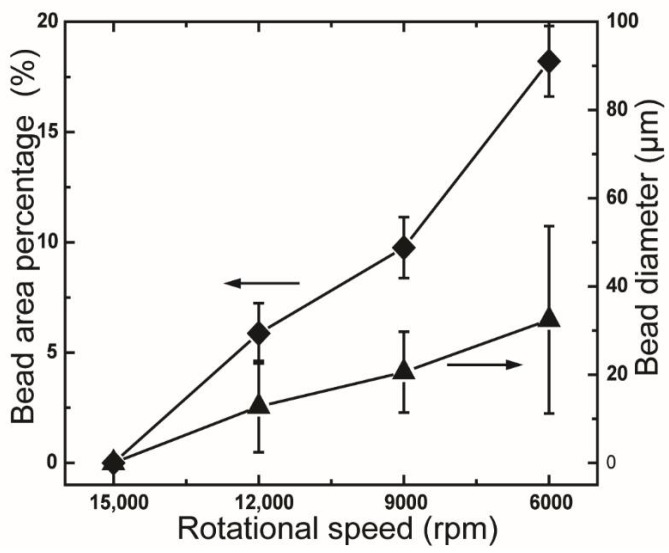
Bead area percentage and bead diameter of rPET mats fabricated under various rotational speeds. Centrifugal spinning condition: polymer solution concentration, 10 wt %; needle inner diameter, 160 μm; collection distance, 10 cm.

Bead-on-string fibers were observed to form when the rotational rate decreased from 15,000 rpm to 12,000 rpm or lower speeds. The bead diameter and bead area percentage of the samples also increased gradually when the used rotational rate was further decreased to 9000 rpm and 6000 rpm. This phenomenon is also due to the occurrence of Rayleigh break-up during the CS process. The decrease in the rotational rate leads to a shorter timescale of surface solidification, which is less than the timescale of Rayleigh instability [28,29]. The effect of the Rayleigh instability induced by the decrement of rotational speed is further evaluated by measuring the bead size and bead area percentage (BAP), as shown in Figure 6. The bead size and BAP are increased from 12.8 μm and 5.8% ± 1.4% to 32.4 μm and 18.2% ± 1.6%, respectively, when the rotation speed is decreased from 12,000 rpm to 6,000 rpm. Moreover, a gradual increase in the bead diameter corresponding to the increase in the rotational rate is also observed, as shown in Figure 6. The SEM images of these samples indicate diameters of 619 ± 235 nm (for 15,000 rpm), 792 ± 287 nm (for 12,000 rpm), 663 ± 269 nm (for 9000 rpm), and 703 ± 282 nm (for 6000 rpm). The highest fiber diameter obtained from fibers at a rotational speed of 12,000 rpm is attributed to the appearance of bead-on-string fibers.

**Figure 7:**
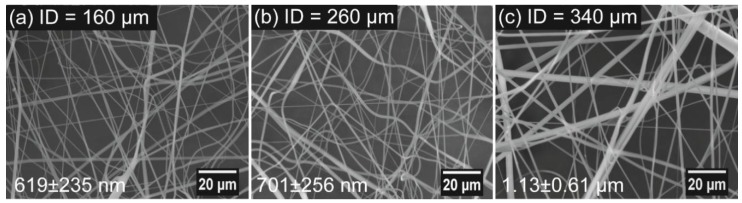
Scanning electron microscope images of centrifugally spun of rPET fibers prepared using needles with various inner diameters: (**a**) 160 μm; (**b**) 260 μm; and (**c**) 340 μm. Centrifugal spinning condition: polymer solution concentration, 10 wt %; rotation speed, 15,000 rpm; collection distance, 10 cm.

The effect of needle size on the morphology and diameter of rPET was also studied by spinning the polymer solution of 10 wt % rPET at a rotation speed of 15,000 rpm using needles with inner diameters (IDs) of 160, 260, and 340 μm. Figure 7 shows SEM images corresponding to needles of various IDs. Figure 7 shows that smooth, detect-free fibers were obtained for all samples fabricated using needles of various IDs. The fiber diameters are 619 ± 235 nm (for ID = 160 μm), 701 ± 256 nm (for ID = 260 μm), and 1.13 ± 0.61 μm (for ID = 340 μm). The increase in the polymer jet diameter induced by increasing the needle ID leads to the largest fibers in the final product. 

The results indicated that the morphology and diameter of centrifugally spun rPET are strongly affected by several processing parameters, including the concentration of the polymer solution (and hence the viscosity) and the rotational speed. These factors are in agreement with published reports, in which the centrifugal spinning process was utilized to fabricate fibers from various kind of polymers such as polycaprolactone [30], poly(ethylene oxide) [31], nylon 6 [32], polyacrylonitrile [23], and poly(vinylidene fluoride) [33]. Defects appear on the fibrous web in the case of low concentration and low rotational speed. Therefore, in order to obtain smooth, uniform fibers, the Rayleigh instability should be prevented by increasing the polymer concentration and the rotational speed.

### 3.3. Mechanical Properties

**Figure 8:**
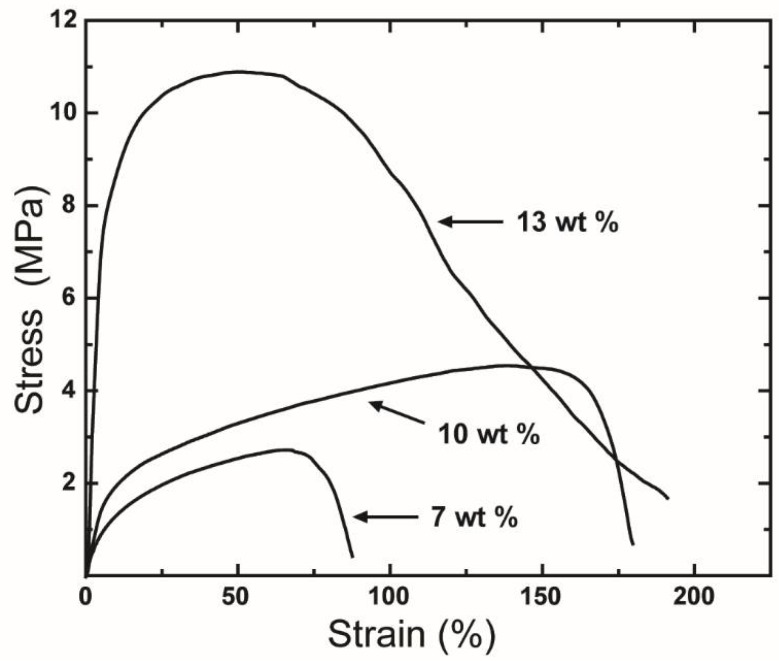
Tensile stress-strain curves of the fiber mats produced from polymer concentrations of 7, 10, and 13 wt %. Centrifugal spinning condition: rotational speed, 15,000 rpm; needle inner diameter, 160 μm; collection distance, 10 cm.

The effect of concentration on the mechanical performance of centrifugally spun rPET fibers was evaluated by measuring the tensile properties of three sets of samples fabricated at rPET concentrations of 7, 10, and 13 wt %. Representative stress-strain curves for these rPET concentrations are shown in Figure 8. The properties of fibers obtained at various polymer concentrations are summarized in Table 1.

As shown in Figure 8, the tensile performance of rPET fibers strongly depends on the polymer concentration. Overall, the mechanical performance of rPET increases as the polymer concentration is increased. The tensile strength, modulus, and strain at the breakage of the rPET fiber web increased when the polymer concentration was increased from 7 wt % to 10 wt % and reached 4.7 ± 0.8 MPa, 33.0 ± 6.8 MPa, and 177% ± 36%, respectively, at 10 wt %. The poor mechanical properties of samples obtained at a polymer concentration of 7 wt % are attributed to the presence of beads on the fiber web. The tensile properties of the fibers produced from a solution at a polymer concentration of 13 wt % are notably improved, with an average strength and an average modulus of 9.2 ± 1.4 MPa and 192 ± 5.1 MPa, respectively, compared to those of the fibrous mats fabricated from polymer concentrations of 7 and 10 wt %. The improvement in the mechanical properties is due to the interconnection between fibers, as discussed in Section 3.2.

**Figure 9:**
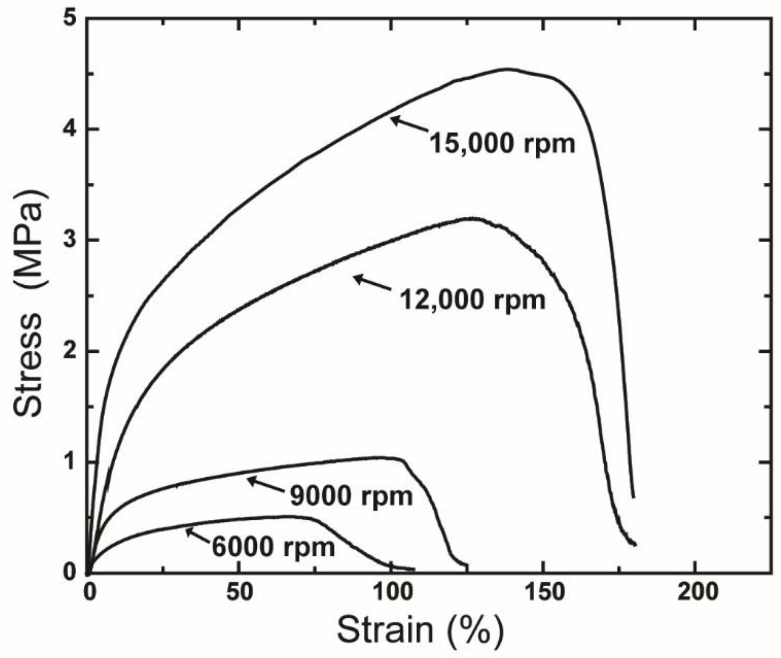
Tensile stress-strain curves of the fiber mats produced under various rotation speeds. Centrifugal spinning condition: polymer solution concentration, 10 wt %; needle inner diameter, 160 μm; collection distance, 10 cm.

The tensile properties of rPET also were influenced by the rotational speed. The tensile stress-strain curves of various centrifugally spun rPET fibers fabricated at various rotational rates are shown in Figure 9, with the detailed mechanical properties of the rPET fibers listed in Table 2.

These results suggest that a decrease in rotation speed leads to a reduction in the tensile performance of fiber mats. The presence of beads on the samples is considered to reduce the mechanical performance of the produced rPET fibers. As the number of beads contained in the fiber web increased, the strength and stiffness were observed to decrease. The variation in tensile strength, modulus, and strain at breakage of products obtained at various rotation speeds is also attributed to the orientation rate of fibers in the direction of the tensile test. At lower speeds, the lower orientation rate of fiber in the circular collectors causes a lower average stiffness and modulus. A decrement in rotation speed also induces a lower orientation rate of polymer chains along the fiber axis, leading to a softer fiber mat.

**Figure 10:**
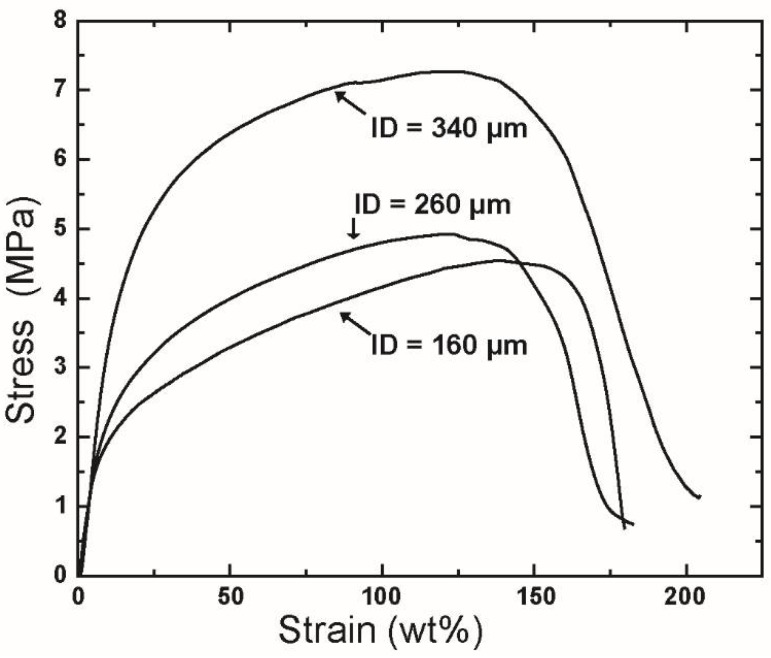
Tensile stress-strain curves of the fiber mats produced using needles with various inner diameters. Centrifugal spinning condition: polymer solution concentration, 10 wt %; rotational speed, 15,000 rpm; collection distance, 10 cm.

The mechanical performance of the resulting fiber was also found to be influenced by the ID of the needles used. The tensile curves of rPET fabricated using needles with various IDs are shown in Figure 10, and the detailed mechanical properties of these fibers are listed in Table 3.

As shown in Table 3, the tensile properties of fibers fabricated from needles having IDs of 160 μm do not appear to change when the ID is increased to 260 μm. This similarity is due to the small change in the fiber diameter of samples generated using these needles, i.e., 619 ± 235 nm for ID = 160 μm and 701 ± 256 nm for ID = 260 μm. However, when the diameter of the fibers increases to 1.13 ± 0.61 μm (for ID = 340 μm), the tensile strength and modulus are increased significantly to 7.8 ± 1.9 MPa and 54.3 ± 15.0 MPa, respectively. The large change in the mechanical properties of the fiber mat produced using needles of ID = 360 μm is attributed to the formation of entanglement between fibers when the fiber diameter is increased.

### 3.4. Thermal Properties

**Figure 11:**
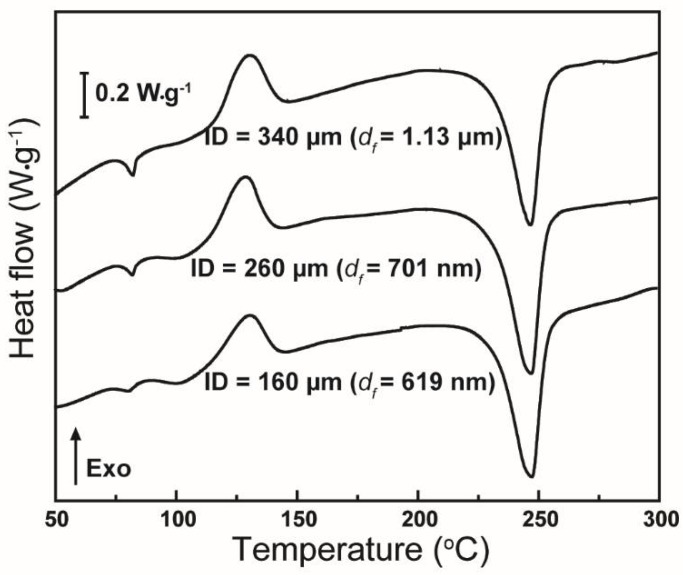
Differential scanning calorimetry thermograms of fibers mats with various average diameters (*d_f_*) produced using needles with various inner diameters. Centrifugal spinning condition: polymer solution concentration, 10 wt %; rotational speed, 15,000 rpm; collection distance, 10 cm.

The DSC heating scans of the as-spun rPET fibers fabricated from the 10-wt % rPET solution at 15,000 rpm using various needles sizes are shown in Figure 11.

Figure 11 shows that the DSC thermograms of centrifugally spun rPET fibers are typical of those expected for PET fibers. The DSC curve of each sample exhibits an enthalpy recovery peak, an exothermic cold crystallization peak, and an endothermic melting peak, which were also observed in electrospun PET [6,34]. The enthalpy recovery peak obtained at around 80 °C is evidence of an oriented amorphous phase (mesomorphic phase) caused by the rapid solidification of stretched PET chains [6,34]. When the diameter of the fibers is increased, the amount of mesomorphic phase is also increased. This increment leads to the higher glass transition temperatures, *T*_g_, of spun rPET fibers, as shown in Table 4. The crystallinity of these sample, as calculated by Equation (2), reveals differences of crystallinity between the different samples, as shown in Table 4. The crystallinity of fibers is 15.1% ± 0.1% (for ID = 160 μm), 13.1% ± 0.6% (for ID = 260 μm) and 11.6% ± 1.3% (for ID = 340 μm). When the fiber diameter is increased (increased ID), the centrifugal force acting on the polymer chains is decreased, leading to an increase in the amount of mesomorphic phase, and thus to a decrease in crystallinity. The effect of polymer concentration on the thermal properties of fibers was also observed, as shown in the Supplementary Materials section (Figure S1 and Table S1). The differences in the thermal behaviors of rPET fibers produced from various polymer concentrations are also attributed to the differences in fiber diameters.

## 4. Conclusions

In conclusion, the CS was successfully used to fabricate recycled poly(ethylene terephthalate) fibrous mats. The effect of polymer concentration and processing parameters on fiber morphology and diameter was investigated. The mechanical properties and the thermal behavior of fibers produced under different conditions were also evaluated. The results implied that the morphology of centrifugally spun fibers was significantly affected by the polymer concentration and the rotation speed. The formation of beads on the fibers and the fiber diameter can be controlled by adjusting the polymer concentration and processing parameters. The results show that the spinnable concentration and rotation speed are above 10 wt % and 15,000 rpm, respectively. The mechanical properties of rPET strongly depend on the experimental parameters. The tensile properties of centrifugally spun rPET fibrous mats increased with the increment of fiber diameter. The DSC curves reveal that the CS process produced an orientated amorphous phase in the rPET fibers, leading to an increase in *T*_g_ of the fibers.

## Figures and Tables

**Table 1:** Mechanical properties of rPET fibers produced from various polymer solutions.

rPET Concentration (wt %)	Mechanical Properties		
	Tensile Strength (MPa)	Modulus (MPa)	Strain at Break (%)
7	3.5 ± 0.7	21.6 ± 4.4	100 ± 20
10	4.3 ± 0.2	34.4 ± 7.1	167 ± 32
13	9.2 ± 1.4	192 ± 56.1	237 ± 74

**Table 2:** Mechanical properties of rPET fibers prepared under various rotation speeds.

Rotational Speed (rpm)	Mechanical Properties		
	Tensile Strength (MPa)	Modulus (MPa)	Strain at Break (%)
15,000	4.3 ± 0.2	34.4 ± 7.1	167 ± 32
12,000	3.5 ± 0.5	18.2 ± 4.7	216 ± 26
9000	1.1 ± 0.1	12.2 ± 5.1	96 ± 20
6000	0.5 ± 0.1	4.3 ± 3.2	92 ± 14

**Table 3:** Mechanical properties of rPET fibers prepared using needles with various inner diameters.

Inner Diameter of Needles (μm)	Mechanical Properties		
	Tensile Strength (MPa)	Modulus (MPa)	Strain at Break (%)
160	4.3 ± 0.2	34.4 ± 7.1	167 ± 32
260	4.5 ± 0.4	32.1 ± 5.1	162 ± 13
340	7.8 ± 1.9	54.3 ± 15.0	205 ± 28

**Table 4:** Thermal properties of rPET fibers prepared using needles having various inner diameters.

Inner Diameter of Needles (μm)	Thermal Properties	
	*T*g (°C)	$\chi_{c}\;$ (%)
160	73.3 ± 1.0	15.1 ± 0.1
260	77.2 ± 0.7	13.1 ± 0.6
340	78.1 ± 0.5	11.6 ± 1.3

## Supplementary Materials

The supplementary materials are available online at http://www.mdpi.com/2073-4360/10/6/680/s1. Figure S1: Differential scanning calorimetry thermograms of fibers mats with various average diameters (df) produced from polymer concentrations of 7, 10 and 13 wt %. Centrifugal spinning condition: rotational speed; 15,000 rpm, needle inner diameter; 160 µm, collection distance; 10 cm, Table S1: Thermal properties of rPET fibers produced from different polymer solutions.
