# Supplementary material for: Centrifugally Spun Recycled PET: Processing and Characterization
Source: Polymers (Basel). 2018 Jun 19;10(6):680. doi: 10.3390/polym10060680 (PMC6404124; doi:10.3390/polym10060680)
Supplement: Supplementary file 1 [file polymers-10-00680-s001.pdf]

Supplementary

Article

Centrifugally Spun Recycled PET: Processing and Characterization

Phu Phong Vo [1,2], Hoan Ngoc Doan [3], Kenji Kinashi [4,*], Wataru Sakai [4], Naoto Tsutsumi [4] and Dai Phu Huynh [2]

[1] Internship Student, Kyoto Institute of Technology, Matsugasaki, Sakyo, Kyoto 606-8585, Japan; 1770195@hcmut.edu.vn

[2] National Key Lab for Polymer and Composite, Faculty of Materials Technology, HoChiMinh City University of Technology, Vietnam National University, HoChiMinh City 700000, Vietnam; hdphu@hcmut.edu.vn (D.P.H)

[3] Doctor's Program of Materials Chemistry, Graduate school of Science and Technology, Kyoto Institute of Technology, Matsugasaki, Sakyo, Kyoto 606-8585, Japan; ngochoandoan@gmail.com

[4] Faculty of Materials Science and Engineering, Kyoto Institute of Technology, Matsugasaki, Sakyo, Kyoto 606-8585, Japan; wsakai@kit.ac.jp (W.S); tsutsumi@kit.ac.jp (N.T)

* Correspondence: kinashi@kit.ac.jp; Tel: +81-075-724-7809.

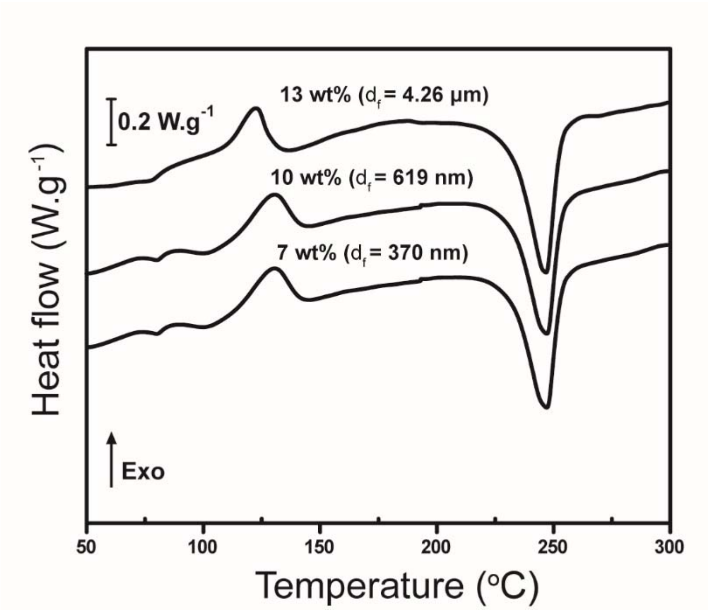

Figure S1. Differential scanning calorimetry thermograms of fibers mats with various average diameters (*d_f*) produced from polymer concentrations of 7, 10 and 13 wt %. Centrifugal spinning condition: rotational speed, 15,000 rpm; needle inner diameter, 160 µm; collection distance, 10 cm.

Table S1. Thermal properties of rPET fibers produced from different polymer solutions.

**rPET concentration (wt %)**	**Thermal properties**	
	**Tg (°C)**	**χ_c (%)**
7	71.1±0.9	17.5±1.2
10	77.2±0.7	13.1±0.6
13	78.5±0.5	11.3±2.3
